# Design and Development of a Scale for Evaluating the Acceptance of Social Robotics for Older People: The Robot Era Inventory

**DOI:** 10.3389/fnbot.2022.883106

**Published:** 2022-07-07

**Authors:** Roberta Bevilacqua, Mirko Di Rosa, Giovanni Renato Riccardi, Giuseppe Pelliccioni, Fabrizia Lattanzio, Elisa Felici, Arianna Margaritini, Giulio Amabili, Elvira Maranesi

**Affiliations:** ^1^Scientific Direction, IRCCS INRCA, Ancona, Italy; ^2^Unit of Geriatric Pharmacoepidemiology, IRCCS INRCA, Ancona, Italy; ^3^Clinical Unit of Physical Rehabilitation, IRCCS INRCA, Ancona, Italy; ^4^Neurology Unit, IRCCS INRCA, Ancona, Italy

**Keywords:** technology acceptance, older people, social assistive robotics, usability, social presence, embodiment, scale validity

## Abstract

**Introduction:**

Nowadays, several robots have been developed to provide not only companionship to older adults, but also to cooperate with them during health and lifestyle activities. Despite the undeniable wealth of socially assistive robots (SARs), there is an increasing need to customize the tools used for measuring their acceptance in real-life applications.

**Methods:**

Within the Robot-Era project, a scale was developed to understand the degree of acceptance of the robotic platform. A preliminary test with 21 participants was performed to assess the statistical validity of the Robot-Era Inventory (REI) scales.

**Results:**

Based on the criteria observed in the literature, 41 items were developed and grouped in different scales (perceived robot personality, human–robot interaction, perceived benefit, ease of use, and perceived usefulness). The reliability of the Robot-Era Inventory scale was analyzed with Cronbach's alpha, with a mean value of 0.79 (range = 0.61–0.91). Furthermore, the preliminary validity of this scale has been tested by using the correlation analysis with a gold standard, the Unified Theory of Acceptance and Use of Technology (UTAUT) model.

**Discussion:**

The Robot-Era Inventory represents a useful tool that can be easily personalized and included in the assessment of any SARs that cooperate with older people in real environment applications.

## Introduction

As stated by the World Population Prospects 2019 (United Nations, 2019), because of the considerable increase in life expectancy, the population of persons aged 80 years or over is thought to triple by 2050. Similarly, the number of people aged over 65 years is rapidly increasing; in 2019, they were 1 in 11, but they will be 1 in 6 by 2050 (UN Department of Economic Social Affairs., 2019). Due to the aging population across the world, a lot of research is being carried out to improve older adults' quality of life and ensure their independence for as long as possible. In this scenario, one of the most explored technological solutions is the use of socially assistive robots (SARs). A social robot is defined as a humanoid or zoomorphic artificial agent. It has been identified as an approach to meeting the mental health needs of older adults through interaction or information exchange (Oh et al., 2018). Despite the increasing interest in the field of assistive robotics and technologies in general, one-third of all the experimented solutions are abandoned during the first year of use (Gurley and Norcio, 2009). For this reason, the design and acceptability of service robots, their ability to positively interact with individuals and coexist in domestic environments, are crucial aspects to overcoming the resistance toward service robotics (Salvini et al., 2010). This topic is frequently explored in literature, confirming that acceptance is often measured qualitatively (Krick et al., 2019). Nevertheless, several scales have been used to evaluate SARs acceptability. The most used is the Technology Acceptance Model (TAM), grounded on the Theory of Reasoned Action (TRA) and the Theory of Planned Behavior (TPB). According to the TAM model, acceptance mainly depends on perceived usefulness and perceived ease of use. These two discussed factors determine the attitude toward use, which, in turn, influences the behavioral intention to use the technology (Ammenwerth, 2019). From the TAM, the Unified Theory of Acceptance and Use of Technology (UTAUT) was derived. The UTAUT model argues that behavioral intention and facilitating conditions influence user behavior. Behavioral intention is, in turn, determined by three constructs: performance, effort expectancy, and social factors. Furthermore, gender, age, experience, and voluntariness of use modulate every factor (Venkatesh et al., 2003). The UTAUT results have been applied to several fields of research, even if neither the TAM nor the UTAUT is specifically validated for the healthcare context (Jewer, 2018) or with older adults (Heerink, 2010). Moreover, some researchers extended the generalization of the original model to the application to patients interacting with SARs (Jewer, 2018). The Almere model is an interesting case of this attempt. This model is founded on the hypothesis that functionality and technological features may not be exhaustive in describing acceptance, but also social dimensions play a crucial role in the acceptance path. Indeed, the Almere model found that trust is moderated by attitude, which, in turn, is moderated by social influence, perceived adaptivity, and anxiety (Heerink, 2010). This model has an enhanced explanatory power, if compared to the original UTAUT (Heerink, 2010), but it was not validated with older users nor did it result theoretically strong, resulting in a limit for the generalizability (de Graaf et al., 2019).

In this article, we report an attempt to provide a comprehensive model and inventory for the evaluation of the Robot-Era platform, developed inside the Robot-Era project (GA 288899). Robot-Era was aimed at developing, implementing, and demonstrating the general feasibility, scientific/technical effectiveness, and social/legal plausibility and acceptability of an advanced social robotic platform, integrated with intelligent environments. The experimental phase of the project was divided into two phases, the first one in a realistic setting and the second one at home. A complete description of the project and publications of the results are available here (https://cordis.europa.eu/project/id/288899/it). After the first experimental phase, the results suggest the need for a more customized tool to assess the acceptability of the Robot-Era platform (Cavallo et al., 2018), as already underlined by relevant authors in this field (Heerink et al., 2009). The preliminary study conducted highlighted the need for a deeper investigation of the social presence dimension, and the abilities relevant to fostering the human–robot interaction (HRI) (Bevilacqua et al., 2015; Cavallo et al., 2018). The Robot-Era Inventory (REI) may represent a first attempt to construct a tool able to include all the metrics of relevance for assessing the acceptability of SARs in the older population, in contrast to the scales already described, and that can be easily personalized based on of the specific services offered. In particular, dimensions, such as usability, social presence, services' acceptability, the personality of the robot, and interaction capabilities, are considered pillars in the field of social robotics assessment, but the relationships among these concepts, the robotic features and human abilities, need to be deeply investigated, to design a model that takes into account the characteristics of the target, i.e., older people, and the peculiarities of the services offered through the robotic solutions and, consecutively, also a tool for measuring and understanding the impact of using SARs.

## Materials and Methods

To build the Robot-Era Inventory, we started with the analysis of the results of the first experimental phase, as clearly described in a study by Cavallo et al. (2018). In light of the results obtained and the literature in the field (Heerink et al., 2008, 2009; Heerink, 2010), the first step was represented by the theoretical design of the model, including all the relevant domains, followed by the drafting of the items to be included in the Inventory. For the development of new concepts for the assessment model of the Robot-Era platform, the starting point of the analysis was represented by the Venkatesh UTAUT model (2003). As the second step, the Robot-Era Inventory was administrated to 21 older people during an experimental setup described in par 2.4, together with the UTAUT questionnaire. The internal validity of the construct of the new scale was evaluated using Cronbach's alpha. The final version of the Robot-Era Inventory is composed of 5 scales.

To build the Robot-Era Inventory, the steps proposed in a study by Boateng et al. (2018) were followed regarding: (a) the Item Development phase (through experts' workshops for identifying the domains and literature reviews on models, tools, and dimensions, described in the following paragraphs) and (b) the Scale Development phase (i.e., pretesting of the items with 21 older participants, first items reduction, and initial analysis of content validity). As the authors already suggest, the steps for scale validation may vary based on the purpose of the study, resources' constraints, and use of existing scales for item generation in contrast with “*de-novo*” tools. For the Robot-Era Inventory, the items were grouped based on already available scales (see Section The Robot-Era Model), plus a customized section related to the Robot-Era platform's services. However, the full validation of the Inventory should include a higher number of participants and a deeper statistical investigation to assess the overall validity and reliability.

### Social Presence and the Human–Robot Interaction

As it is well known from the literature (Lee et al., 2006; Heerink et al., 2008), social presence can be considered a determinant of the acceptability and usability of socially assistive robots. This particular dimension has received much interest both in the field of social psychology and human–robot interaction (Biocca et al., 2004). Many definitions of this concept have emerged, but it can be said that the term “social presence” can be referred to as “the sense of being there” (Witmer and Singer, 1998; Biocca, 2004) or the feeling of being in the company of someone as “the perceptual illusion of nonmediation” (Lombard and Ditton, 1997). From the psychological perspective, the social presence can be ascribed to the “theory of mind” paradigm (Gordon, 1986; Carruthers and Smith, 1996). Following this theory, it can be said that when interacting with a robot, the users expect the robot to respond socially, to be able to express affection and appropriate responses to the person's social input, and, thus, to stimulate emotional reactions (Damiano et al., 2015). In this way, it is possible to assess the social presence of a SAR, by determining how the robot can interpret social stimuli and how humans perceive and interpret the robot socially (Fiore et al., 2013).

In 2004, Lee classifies three types of presence:

- The physical experience of entities or environments.
- The social experience refers to the experience of social actors (both the humans and human like).
- The self-experience refers to the experience of one's self or selves.

Out of the three types, the social presence plays a crucial role for the human–robot interaction and it could be considered the ultimate goal of any designer of SARs (Breazeal, 2003; Fong et al., 2003; Lee and Nass, 2003). It is, therefore, important that through social signals, the robot conveys its social presence (Fiore et al., 2013), to allow the person to consider the robot as a social agent, able to influence the sociocognitive processes of the individuals (Biocca and Harms, 2002; Fiore et al., 2013). Finally, Biocca et al. (2004) suggested the need to contextualizing the theory of social presence and its measurement, matching the insights from the literature with the research objectives.

As for humans, the communication “rules” should guide the development of social robots defined as “an autonomous or semi-autonomous robot that interacts and communicates with humans” (Bartneck and Forlizzi, 2004). In fact, to date, robots are not seen as simple tools anymore, but also as companions, thus able to interact socially with humans (Cobo Hurtado et al., 2021). To do that, the robot must be able to understand what the user is saying or doing, understand natural language, and should be capable of establishing complex dialogs with its human. As described also by Fong et al. (2003), the social robots should have the following characteristics:

- Express and/or perceive emotions
- Communicate with high-level dialog
- Learn/recognize models of other agents
- Establish/maintain social relationships
- Use natural cues (gaze, gestures, etc.)
- Exhibit distinctive personality and character
- May learn/develop social competencies.

Responsiveness and prompt support are mostly requested in emergency conditions, in which the user expects to receive coherent and rapid feedback on the circumstance, to act appropriately. For this purpose, the HRI may be supported by multichannel sensory features, which generally include auditory, visual, and tactile capabilities. Also, the esthetical parts of the robots are relevant for communication, such as eyes, dimensions, and shape (Bonarini, 2020). To appreciate and measure the quality of the HRI, key elements of the interaction should be defined during the setup of any experimentation or the design of a new product: the human, the robot, their interaction, and the context (Collins, 2019).

Regarding human characteristics, five personality traits of the user are strong predictors of a positive HRI, namely, extroversion, agreeableness, conscientiousness, neuroticism, and openness to experience (Esterwood and Robert, 2021).

### Acceptability

The acceptance of technology represents more complex phenomenon with respect to the analysis of older people's needs *per. se* and it could be defined as “the demonstrable willingness within a user group to employ technology for the task it is designed to support” (Mynatt et al., 2000; Al-Youssef, 2015).

In general, there is a tendency to think that older adults are less interested in technological advances and the use of technology (Knapova et al., 2020). To understand the kernel of the older people, rejection of new technological artifacts means to understand deeply the person beliefs that characterized the elderly and that can determine their closure to the innovation. Although there are advantages to the use of technology by older people, it is possible to notice a rejection of the artifacts, caused by the low motivation to use technology, little knowledge about the computer/technological world, and also the cognitive and physical changes that older people undergo as they age (Wildenbos et al., 2018). This last factor specifically leads to a psychological condition known as “technostress,” a construct that indicates how the difficulty of older people in using technology leads to anxiety and depression about technology and, therefore, a low level of acceptance of it (Nimrod, 2018).

The acceptance of a device is linked to intrinsic or extrinsic factors related to the technology (Flandorfer, 2012), such as living environments, social relationships, and needs, and it may lead to the diffusion and exploitation of the systems, supporting new markets and discovering new segments of consumers.

There are numerous studies (Wagner et al., 2010; Magsamen-Conrad et al., 2015; Vroman et al., 2015; Knapova et al., 2020; Zaman et al., 2022) that have researched and identified factors that explain the level of technology acceptance by older people. Personal factors, such as age and education level (Magsamen-Conrad et al., 2015; Vroman et al., 2015; Vorrink et al., 2017), psychological factors, such as motivation to use technology, perceived anxiety, and cognitive abilities (Venkatesh et al., 2003; Macedo, 2017), and environmental factors, such as financial support and assistance from friends and family (Wagner et al., 2010), come into play. Finally, personality-related factors also play an important role (Vroman et al., 2015).

In this regard, Svendsen et al. (2013) have investigated the degree to which users' assessment of the core constructs of the Technology Acceptance Model (TAM) is influenced by personality as measured by a short version of the Big Five Inventory (John et al., 1991). A web-based survey was used where 1,004 users read a description of a software tool before completing personality and the TAM inventories. The results indicate that personality influences behavioral intention (BI). In particular, the extraversion trait has significant, positive relations to BI and this relation is fully mediated by the TAM beliefs, in addition to the openness to experience, significantly and positively related to perceived ease of use.

In this case, the analysis of the acceptability of the three robotic platforms was mainly based on the administration of the UTAUT questionnaires and *ad-hoc* questions on anxiety and perceived enjoyment, and the evaluation of the acceptability oriented to the services, employing through observations. Moreover, the analysis of two personality traits, namely, the novelty-seeking and the introversion/extraversion traits, will be added to the preliminary questionnaire, while Anxiety, Attitude, and Perceived Adaptability scales from the UTAUT were selected. Among them, it was found that perceived adaptability is a crucial dimension for evaluating the acceptance of social robots in older people (Heerink et al., 2008).

### Usability

Older people are often considered “technophobes” due to their scarce knowledge and lack of accessibility to technology (Joshi et al., 2020). The use of a robotic assistant for daily activities can be felt by older people as a real challenge. Furthermore, long-term use of robots is also rare because little research has tested them in real human operating environments, where both the needs and difficulties of interaction emerge (Cobo Hurtado et al., 2021).

Understanding the role of usability in the field of robotics is not trivial, as the technical features of the robots are inextricably connected with factors, such as social presence, empathy, and feeling of being in a relationship (Rogers, 2009).

Following the principles of universal design, a product and an environment should be usable by all the people, avoiding, as much as possible, the need for adaptation or specialized design, through the application of principles, such as equitable use, flexibility, simplicity, and intuitive use, perceptible information, tolerance for error, low physical effort, and size and space for approach (Burgstahler, 2001). This means that the technology should be built for as wider a range of users as possible and also for secondary and tertiary end-users, most of all for the informal caregivers (Van Den Broek et al., 2010). Technological malfunctioning and limitations of robots represent two of the most important barriers to the adoption of social robots. Moreover, it can be stated that the usability of a robotic system is a major concern among older adults (Papadopoulos et al., 2020).

Robots are smart objects that can be distinguished from other similar products due to their navigation and manipulation skills, in addition to the interaction modality. The usability is influenced strongly by interactions that are executed by hardware and moving parts, not only by software. Robots can move around autonomously, they can interchange or manipulate objects with users, and due to their stronger interaction skills, they can be not only perceived as machines, but also personal assistants or even friends. For this reason, it was decided to maintain the concepts expressed by ISO 9241 and the UTAUT dimensions of perceived usefulness and perceived ease of use as relevant references for the usability evaluation, both largely described in the literature.

### Robot-Era Model

The Robot-Era model is designed in light of the literature in the field and the lessons learned from the first testing experience with the Robot-Era platform. In particular, the adaptation of available tools seemed necessary to include a more comprehensive approach to social presence and the HRI, determined by the robotic capabilities and characteristics.

The relevant factors behind the model are divided into: *intrinsic. characteristics* and *interaction. factors*.

For *intrinsic. characteristics*, we defined all those end-users and robots' characteristics that influence the interaction condition, determined by embodiment and social navigation for the robotic agent and acceptability antecedents, such as attitude toward technology, personality traits, age, gender, technology representations, eHealth, and health literacy, for the human agent.

Regarding the robotic agent, the embodiment and social navigation establish a basis for structural coupling by creating the potential for mutual perturbation between system and environment, a prerequisite for any robotic agent to be perceived as a social being (Fong et al., 2003). On the human agent, the acceptability antecedents represent a core set of essential information to be collected, as largely reported in many studies and theoretical approaches in the field (Bevilacqua et al., 2014).

As *interaction. factors*, we have defined all the key dimensions to assess the overall acceptability of social robotics in any experimental setup that required contact with older people to cooperate in daily activities. These factors are:

- *Perceived. robot. personality*, including all the characteristics related to social presence, trust, and feeling of being in company with someone.
- *Human–robot. interaction*, including the assessment of communication skills and speech, perceived safety, and physical contact.
- *Usability*, intended as ease of use and perceived usefulness.
- *Acceptability*, intended as attitude toward the system (in this case, the Robot-Era, but these should be customized based on the robotic services or technology), perceived benefit, and adaptability to the needs and wishes of the participants.

**Figure 1:**
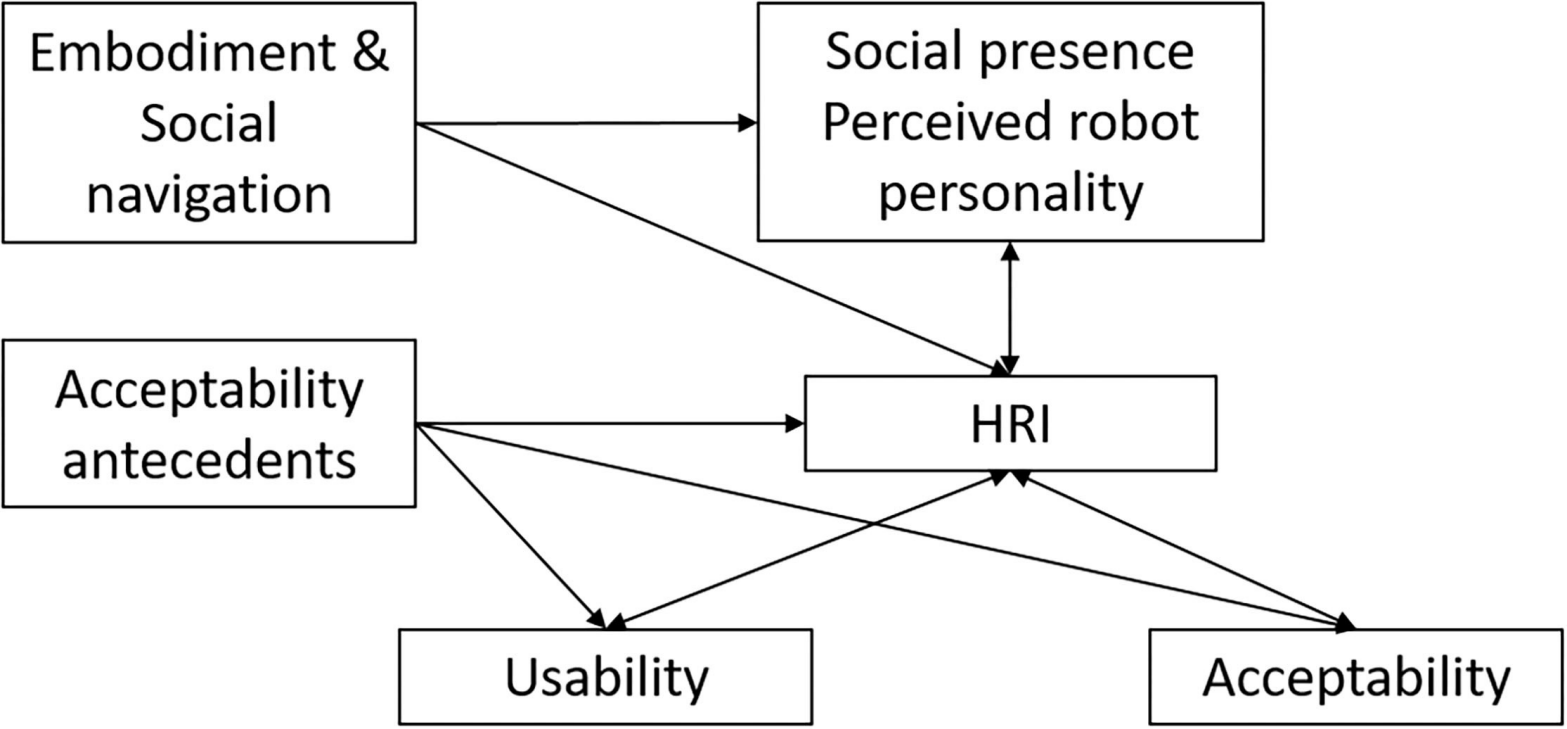
The Robot-Era theoretical model that summarized the direct influences within the different factors.

Even if all the dimensions are strictly connected, Figure 1 summarizes the direct influences of the different factors: the robotic agent's intrinsic characteristics, for example, directly influence the social presence and the HRI capabilities, while the human agent's acceptability antecedents may have a direct effect on the perception of usability and acceptability of SAR, and on the evaluation of the HRI features themselves. As observed, the HRI plays a central role in the model, as it is the domain in which the robotic agent and the human agent' dynamics converge together, in the co-construction of the social interaction.

To draft the items of the inventory, the tools and scales from the literature were selected (Interpersonal Attraction Scale; McCroskey and McCain, 1974; UTATU, Venkatesh et al., 2003; Godspeed questionnaire, Bartneck et al., 2009) and then customized for the Robot-Era robotic services. Table 1 shows the items selected for the inventory.

**Table 1:** Items selected for the inventory.

**Dimension**	**Acronym**	**ITEMS**
Perceived Robot Personality	PRP	The robot is unsociable-sociable The robot is insensitive-sensitive The robot is incompetent-competent The robot is unintelligent- intelligent The robot is moving rigidly- moving elegantly The robot could be a friend of mine I would like to have a friendly chat with the robot I would trust the robot if it gives me advice I have confidence in the robot ability to get the job done I'm afraid the robot can hurt me I feel safe when the robot moves around me
Human Robot Interaction	HRI	The robot was able to communicate his intention clearly to me When talking with the robot I felt like I'm talking to a real person The vocabulary of the robot is appropriate The robot talks fluently The robot is able to manage communication failures How do you feel when the robot was moving his arm? agited-calm How do you feel when the robot was moving his arm? quiescent-surprise How do you feel when the robot speech? agited-calm How do you feel when the robot speech? quiescent-surprise I think talk to the robot is very easy
Perceived Benefit	PB	The robot is appealing and I really would like to use it more I do not have the technical competences to make a good use of the robot I think I could have a good use of the robot Robot services match the needs I have The robot is able to fulfill the goal I have settled I feel more independent if supported by the robot in my daily activities
Easiness of use	EU	I couldn't get anything accomplished with the robot I will be able to use the robot without any support I think the overall RE platform can be used only by people with no limitation I have had fun using the robot I was relaxed during the use of the robot I feel nervous while using the robot
Perceived usefulness	PU	Reminding appointment Communicate with carers Carrying objects Giving the sense of security in the home Accompany inside the homeh36pay I could use Robot-Era system only if necessary I am willing to my living environment to be able to use the robot.

### Sample Description and Procedure

To assess the internal validity of the Robot-Era Inventory, a preliminary test with 21 participants was performed, to be replicated with a larger sample in a more advanced stage, in case of initial positive evidence.

#### Sample Description

The study population consisted of 21 volunteers from a local recreational center of the municipality, 13 men and 8 women, with a mean age of 69.2 ± 3.9 years. Information about their educational level, working situation, and monthly income are given in Table 2.

**Table 2:** Sample description.

**Variable**
Age, mean ± SD	69.2 ± 3.9
**Gender**, ***n*** **(%)**
Male	13 (61.9%)
Female	8 (38.1%)
**Educational level**, ***n*** **(%)**
Primary	9 (42.9%)
Secondary	7 (33.3%)
Tertiary	5 (23.8%)
Education in years, mean ± SD	11.2 ± 4.1
**Working situation**, ***n*** **(%)**
Retired	16 (76.2%)
Working full time	2 (9.5%)
Working at home	2 (9.5%)
**Monthly income**, ***n*** **(%)**
0–500 €	1 (5.3%)
501–1,000 €	2 (10.5%)
1,001–1,500 €	7 (36.8%)
1,501–2,000 €	6 (31.6%)
2001–2500 €	3 (15.8%)

#### Procedure

The first version of the Robot-Era Inventory was composed of 41 items to be rated on a 5-point Likert scale. The statistical validation of the inventory was conducted in a piloted session in the IRCCS INRCA facility with 21 older people, to test the validity of the scales concerning the goal standard scale (the UTAUT questionnaire). After the presentation of Robot-Era objectives and the main functionalities and characteristics of the three robots, the researchers have shown a video of Robot-Era platform operating in indoor and outdoor contexts, to give a concrete idea of the potential use of the robots. Before starting the test, informed consent was signed by each participant and the subjects' anonymity was guaranteed. After they saw the video, the Robot-Era Inventory and the UTAUT questionnaire were administrated. This video may be found here: https://www.youtube.com/watch?v=XVJXdIZ6GVA.

### Statistical Analysis for the Development of the Robot-Era Inventory

The reliability of the Robot-Era Inventory scales was analyzed with Cronbach's alpha. Cronbach's alpha is a coefficient of internal consistency reliability and a solid construct would have an alpha of at least 0.7 (Nunnally, 1978). The interpretation of Cronbach's alpha is (Gliem and Gliem, 2003): *a*. ≥. 0.9 Excellent; 0.8 ≤ *a*.<. 0.9 Good; 0.7 ≤ *a*.<. 0.8 Acceptable; 0.6 ≤ *a*.<. 0.7 Questionable; 0.5 ≤ *a*.<. 0.6 Poor; and *a*.<. 0.5 Unacceptable. Based on the criteria observed in the literature and the analysis, 41 items were developed and grouped in different scales (Table 1). In addition, the same analysis was performed on the UTAUT results to verify the reliability of the scale in the experimental setting. Finally, the internal validity of the constructs has been tested using the correlation analysis between the Robot-Era Inventory and the Unified Theory of Acceptance and Use of Technology (UTAUT) (Venkatesh et al., 2003) subscales. The Bonferroni correction has been applied to correct the multiple comparisons. A confirmatory principal component analysis (PCA) using Varimax rotation method with Kaiser normalization is conducted.

## Results

Cronbach's alpha values of the Robot-Era Inventory (REI) scales and the UTAUT scale are given in Tables 3, 4, respectively. In Table 3, an adequate level of reliability is shown by the scores that are all > 0.6. Table 4 shows that the UTAUT scales also have good internal consistency within this study, except social influence (SI). The correlation coefficients, after the Bonferroni correction, between the Robot-Era Inventory scales and the UTAUT scales are shown in Table 5. As expected, there is a high value of the correlation coefficients between the REI and UTAUT subscales, even if only some correlations are significant. Several UTAUT subscales do not show a significant correlation coefficient with the REI subscales, probably due to the low sample size. Two of the most important subscales in the literature and also in our model are the TRUST and PS subscales. Both have a positive and significant correlation with all of the HRI and PB subscales, respectively. In fact, in both the models, importance is placed on the robot's ability to interact with the person. However, the limitation of the UTAUT is that it was not built for the elderly person, as opposed to the REI. Another important scale is the intention to use (ITU), which is considered an essential scale for technology usage adoption. In our case, it has two correlations with our inventory with the perceived usefulness and with the human–robot interaction.

**Table 3:** The Cronbach's alpha values of the Robot-Era Inventory scales.

**Dimension**	**Acronym**	**Cronbach's Alpha**
Perceived Robot Personality	PRP	0.7416
Human Robot Interaction	HRI	0.6947
Perceived Benefit	PB	0.8480
Easiness of use	EU	0.6784
Perceived usefulness	PU	0.7064

**Table 4:** The Cronbach's alpha values of the Unified Theory of Acceptance and Use of Technology (UTAUT).

**Subscale**	**ACRONYM**	**Cronbach's Alpha**
Anxiety	ANX	0.9269
Attitude	ATT	0.8341
Facilitating conditions	FC	0.8627
Intention to use	ITU	0.9305
Perceived adaptability	PAD	0.7386
Perceived enjoyment	PENJ	0.9232
Perceived ease of use	PEOU	0.6393
Perceived usefulness	PU	0.8369
Social influence	SI	0.4942
Trust	Trust	0.8773
Social presence	SP	0.8846
perception of sociability	PS	0.8909

**Table 5:** Correlation coefficients with the Robot-Era Inventory scales and the UTAUT scales after the Bonferroni correction.

	**REI**				
		**PRP**	**HRI**	**PB**	**EU**	**PU**
UTAUT	**ANX**	−0.3306	−0.4631	−0.4630	0.0142	−0.3617
	**ATT**	0.4618	0.5797	0.5046	0.3914	0.6134
	**FC**	0.2738	0.5755	0.5165	0.433	0.5552
	**ITU**	0.6865	0.7454*	0.5497	0.4498	0.8175*
	**PAD**	0.5301	0.7392*	0.6647	0.3933	0.5345
	**PENJ**	0.5166	0.7654*	0.5173	0.4665	0.3527
	**PEOU**	0.3548	0.6693	0.6404	0.6857	0.6621
	**PU**	0.3024	0.4866	0.5566	0.6427	0.6507
	**SI**	0.3973	0.2222	0.2311	−0.0265	0.3135
	**Trust**	0.6042	0.7479*	0.6952	0.5511	0.5700
	**SP**	0.6619	0.5817	0.6304	0.3305	0.5869
	**PS**	0.6166	0.5070	0.7678*	0.5892	0.6496

*ANX, Anxiety; ATT, Attitude; FC, Facilitating conditions;ITU, Intention to use; PAD, Perceived adaptability; PENJ, Perceived enjoyment; PEOU, Perceived ease of use; PU, Perceived usefulness; SI, Social influence; Trust, Trust; SP, Social presence; PS, Perception of sociability; PRP, Perceived Robot Personality; HRI, Human Robot Interaction; PB, Perceived Benefit; EU, Easiness of use; PU, Perceived usefulness*.

^*^=r < 0.05.

Table 6 reports the analysis of the sociodemographic characteristics of the sample, concerning the score obtained on the REI and the UTAUT scales. As it is observed, no significant differences in age, class, and gender were found for the REI scales, while for the UTAUT, there is a significant positive correlation between gender and facilitating condition, suggesting a positive perception of available personal resources to use the robot from the male respondents, and a higher perception of social influence for the older people, underlying a probable positive role of the environment to foster the system acceptability.

**Table 6:** The Robot-Era Inventory scales and the UTAUT by the gender and age groups, mean ± SD.

**Scales**		**Total**	**Gender**			**Age group**		
			**Male**	**Female**	**p**	**<70**	**70+**	**p**
**REI**	**PRP**	37.6 ± 5.7	37.8 ± 6.3	37.4 ± 5.2	0.883	37.3 ± 5.3	37.1 ± 6.4	0.938
	**HRI**	34.9 ± 4.5	36.2 ± 3.4	32.8 ± 5.5	0.088	35.0 ± 4.7	35.0 ± 4.8	0.999
	**PB**	22.8 ± 4.9	23.5 ± 5.5	21.6 ± 3.7	0.398	22.9 ± 3.4	21.8 ± 6.5	0.903
	**EU**	17.7 ± 2.4	18.4 ± 2.0	16.6 ± 2.8	0.106	17.5 ± 2.8	17.5 ± 1.3	0.999
	**PU**	28.9 ± 4.0	29.2 ± 3.9	28.4 ± 4.2	0.642	29.2 ± 3.7	27.6 ± 3.8	0.376
**UTAUT**	**ANX**	9.2 ± 5.0	8.5 ± 4.9	10.5 ± 5.3	0.379	9.8 ± 4.9	9.0 ± 5.5	0.725
	**ATT**	11.0 ± 2.5	11.5 ± 2.0	10.3 ± 3.1	0.290	11.3 ± 2.0	10.0 ± 2.8	0.228
	**FC**	6.4 ± 1.9	7.2 ± 1.3	5.0 ± 2.2	**0.013**	6.3 ± 1.7	6.3 ± 2.4	0.989
	**ITU**	7.8 ± 3.1	8.3 ± 2.9	6.9 ± 3.5	0.337	8.4 ± 3.1	6.5 ± 2.8	0.198
	**PAD**	10.2 ± 2.4	10.8 ± 1.8	8.9 ± 3.0	0.075	10.4 ± 2.3	9.6 ± 2.6	0.526
	**PENJ**	17.7 ± 2.7	18.3 ± 2.5	16.9 ± 3.1	0.295	17.9 ± 2.7	17.6 ± 3.2	0.811
	**PEOU**	17.3 ± 3.9	18.4 ± 2.1	15.5 ± 5.4	0.097	17.2 ± 3.5	17.0 ± 4.7	0.928
	**PU**	10.3 ± 2.6	11.1 ± 2.1	9.0 ± 2.9	0.071	10.3 ± 2.7	9.9 ± 2.2	0.751
	**SI**	8.1 ± 1.9	8.1 ± 2.1	8.2 ± 1.6	0.940	9.0 ± 1.0	7.0 ± 2.4	**0.038**
	**Trust**	7.2 ± 2.1	7.6 ± 2.0	6.5 ± 2.3	0.250	7.5 ± 2.2	6.6 ± 2.1	0.389
	**SP**	11.8 ± 4.3	13.0 ± 4.5	9.9 ± 3.2	0.130	10.8 ± 4.0	12.9 ± 4.9	0.353
	**PS**	11.9 ± 4.9	12.5 ± 4.9	10.9 ± 4.9	0.479	11.3 ± 4.8	12.1 ± 5.3	0.719

*p-values from unpaired t-test*.

*ANX, Anxiety; ATT, Attitude; FC, Facilitating conditions; ITU, Intention to use; PAD, Perceived adaptability; PENJ, Perceived enjoyment; PEOU, Perceived ease of use; PU, Perceived usefulness; SI, Social influence; Trust, Trust; SP, Social presence; PS, Perception of sociability; PRP, Perceived Robot Personality; HRI, Human Robot Interaction; PB, Perceived Benefit; EU, Easiness of use; PU, Perceived usefulness*.

*Bold values indicated statistically significant differences*.

The factor loading obtained from the confirmatory principal component analysis is reported in Supplementary Materials. This analysis confirms the validity of the theoretical subdivision in subscales reported in this article. The PCA showed 5 components: the first component corresponds to perceived benefit, the second component corresponds to perceived robot personality, the third component corresponds to the human–robot interaction, and the fifth component corresponds to perceived usefulness. The second component is the only one that has not immediate correspondence with our model. Correlations among PCA components and the REI subscales are tested with Pearson's coefficients, which range from 0.3751 for factor 4 to 0.8807 (*p* < 0.05) for factor 1 corresponding to the REI PB.

## Discussion

Given the rising number of older people in nowadays society, it is essential to understand the acceptability of social robotics to support them in daily activities. The pervasiveness of robotics in the healthcare context requires a deeper analysis in terms of impact on the quality of life and cost-effectiveness of the innovative solutions, but the successful diffusion of such devices is strongly determined by their acceptability, in the short and long term.

In literature, it is widely recognized the paramount importance of the TAM and the UTAUT models, aimed at providing insights on how to support the use of innovative systems, especially robotics in the latter case. However, the authors suggest the need to adapt the model and the questionnaires to the requirements of the experimental setting, and the technological artifacts (Heerink et al., 2008). Moreover, as the research in the field is becoming more and more multidisciplinary, understanding the impact of technology acceptance on the quality of life of older people is a central topic, especially for geriatricians. However, there is still limited evidence of tools that assess the perceived improvement of the quality of life, in combination with the acceptance of technological services. This limitation is also due to the use of qualitative methods and/or clinical scales in technological trials, such as the Short Form-12, for example, to address the improvement of quality of life after the system use. These tools are designed for the clinical population in assistance and care settings (Ware et al., 1996) and not to understand the impact of technology for supporting active and healthy aging, for example, at home, as they include the assessment of a wide range of dimensions that are not the target of technological devices, as SARs. The same can be said for the independent living and autonomy domains. In this case the most used tools are activities of daily living and instrumental activities of daily living indexes (Lawton and Brody, 1969). These scales are designed by adopting a medical perspective to assess the functional and cognitive autonomy of older people, but those activities (i.e., dressing, bathing, managing, financing) are only partially addressed by the robotic solutions and require a more complex combination of technological and personal assistance to be supported. There are wider concepts and definitions of autonomy in aging that may open up to a profound understanding of the impact of technology and its acceptance, not only of the aging phenomenon. As the objective of any technological tool is the promotion of an optimal aging process, the definition of successful aging has the achievement of “high physical, psychological, and social functioning in old age without major diseases” (Fries, 1980; Cosco et al., 2014; Martin et al., 2014; Bevilacqua et al., 2020), seems to be more appropriate to unveil the activities that the older people consider of utmost importance for their quality of life and that may be supported through technologies. More recently, intending to promote a more comprehensive and appropriate assessment of the aging population, the WHO introduced the concept of intrinsic capacity (IC), defined as “the composite of all the physical and mental capacities that an individual can draw upon during his/her life” (Beard et al., 2016), open up to those intrinsic characteristics that the older people can put in place during the aging process and that play a crucial role in the technology acceptance and usage behavior.

In our model, we have tried to combine a wider approach to understand the impact on the quality of life of the personalized robotic services offered through the Robot-Era system, with the construct of acceptance of technology from a traditional model, like UTAUT. The Robot-Era Inventory includes the assessment of the personalized services, an adaptation already suggested in the literature (Heerink et al., 2008), with the evaluation of the perceived robotic capabilities, influenced by the end-users intrinsic characteristics. As it was designed, the dimension of the human–robot interaction (HRI) represents the kernel of the model, by including the evaluation of the robotic capabilities (i.e., speech) and the perception of those by the older users, representing a co-constructed space between the two agents that shape the relationship, influencing the use of the system.

This study represents an attempt to take a step further in the field of technology assessment with older people, concerning the SARs, and also a solution to the urgent need for the availability of customizable tools, to be adapted to different experimental setups and services.

As psychophysiological measures are considered one of the main methods of assessment used for human studies in the human–robot interaction together with self-report, behavioral measures, and task performance analysis (Bethel et al., 2007), future studies should take into consideration the use of the scale of acceptance in combination with biosignals, for example, related to anxiety during the use of the robot. As the physiology of the autonomic nervous system changes with age, the comprehension of the autonomic arousal concerning to stressful stimuli, such as the use of a SAR, is of paramount relevance in combination with traditional assessment tools, to understand the reactions of older people during the performance with the technology. Several studies on social robotics have used combined evaluation of quantitative and/or qualitative tools with biosignals, such as ECG, electrodermal activity, and the electric brain activity, with the aim of personalizing the behavior of the robot concerning to the emotional state of the older users (Fiorini et al., 2020).

Despite this, this study presents some limitations. First of all, a higher number of participants should be involved in the scale assessment, to collect data to refine the inventory. A shorter version of the inventory needs to be developed to be applied during any experimental setting, so as not to constitute a burden for the older respondents. Moreover, despite the validity of the video analysis as the methodology to evaluate the HRI, the opportunity of administrating the questionnaire after an effective interaction in a real or realistic setting can be relevant. In the future, a cultural validity of the inventory, by including older volunteers from different cultural backgrounds and equally divided by gender, should be conducted.

## Data Availability Statement

The raw data supporting the conclusions of this article will be made available by the authors, without undue reservation.

## Ethics Statement

Ethical review and approval was not required for the study on human participants in accordance with the local legislation and institutional requirements. The patients/participants provided their written informed consent to participate in this study.

## Author Contributions

EM and RB: study concept and design. RB and EF: acquisition of data. RB and MD: analysis and interpretation of data. EM, RB, and MD: drafting of the manuscript. GP, GR, and FL: critical revision of the manuscript for important intellectual content. AM and GA: writing—reviewing and editing. All authors have read and agreed to the published version of the manuscript.

## Funding

This study was conducted for the Robot-Era project, funded by European Commission under grant agreement 288899.

## Conflict of Interest

The authors declare that the research was conducted in the absence of any commercial or financial relationships that could be construed as a potential conflict of interest.

## Publisher's Note

All claims expressed in this article are solely those of the authors and do not necessarily represent those of their affiliated organizations, or those of the publisher, the editors and the reviewers. Any product that may be evaluated in this article, or claim that may be made by its manufacturer, is not guaranteed or endorsed by the publisher.
