# Supplementary material for: Design and Development of a Scale for Evaluating the Acceptance of Social Robotics for Older People: The Robot Era Inventory
Source: Front Neurorobot. 2022 Jul 7;16:883106. doi: 10.3389/fnbot.2022.883106 (PMC9302437; doi:10.3389/fnbot.2022.883106)
Supplement: Supplementary file 1 [file Table_1.docx]

Factor loadings of the confirmatory principal component analysis (PCA) using Varimax ration method with Kaiser normalisation

| **Variable** | **Comp1** | **Comp2** | **Comp3** | **Comp4** | **Comp5** |
| --- | --- | --- | --- | --- | --- |
| I'm afraid the robot can hurt me | 0.0805 | -0.1941 | 0.2168 | -0.1402 | 0.2256 |
| I feel safe when the robot moves around me | -0.0301 | 0.0025 | 0.0051 | 0.2482 | 0.2592 |
| I was relaxed during the use of the robot | 0.1558 | -0.0279 | 0.0067 | -0.1171 | 0.3055 |
| I couldn't get anything accomplished with the robot | 0.0875 | -0.2335 | 0.1283 | -0.0016 | -0.2502 |
| communicate with carers | 0.1634 | 0.0697 | -0.0387 | 0.1264 | 0.2048 |
| carrying objects | -0.0118 | 0.0405 | -0.0278 | 0.1634 | 0.2791 |
| giving the sense of security in the home | 0.2135 | -0.023 | 0.0275 | 0.0794 | 0.2219 |
| accompany inside the home | 0.091 | 0.0189 | 0.0098 | -0.046 | 0.3287 |
| In order to use Robot-Era system, I think I could pay a price for it | 0.2046 | 0.1139 | 0.0271 | -0.1181 | -0.314 |
| I could use Robot-Era system only if necessary | 0.0059 | -0.0677 | 0.2435 | -0.1098 | 0.3083 |
| I would trust the robot if it gives me advice | 0.1642 | 0.0501 | 0.0705 | 0.2841 | -0.1673 |
| I have confidence in the robot ability to get the job done | -0.0939 | 0.0073 | -0.0503 | 0.3495 | 0.0828 |
| The robot was able to communicate his intention clearly to me | 0.0924 | -0.0093 | -0.0735 | 0.1619 | 0.0455 |
| The robot is able to manage communication failures | 0.0367 | 0.0149 | 0.1889 | 0.336 | -0.0948 |
| I feel more independent if supported by the robot in my daily activities | 0.2322 | 0.1362 | 0.0886 | -0.2385 | -0.0029 |
| I will be able to use the robot without any support | 0.1168 | -0.0358 | -0.0703 | 0.3549 | -0.0383 |
| reminding appointment | -0.0395 | 0.0335 | 0.0216 | 0.3771 | 0.0552 |
| The vocabulary of the robot is appropriate | 0.1243 | 0.0313 | 0.2531 | 0.0781 | -0.1284 |
| How do you feel when the robot was moving his arm? agited-calm | 0.0743 | 0.0039 | -0.3504 | 0.0638 | -0.0097 |
| How do you feel when the robot speech? agited-calm | 0.1091 | -0.0821 | -0.3796 | 0.0145 | -0.0267 |
| How do you feel when the robot speech? quiescent-surprise | -0.0008 | 0.1737 | -0.3376 | -0.0771 | -0.1144 |
| I think the overall RE platform can be used only by people with no limitation | -0.0192 | 0.1008 | 0.2502 | 0.0068 | -0.0178 |
| I feel nervous while using the robot | 0.0222 | -0.0906 | 0.2076 | -0.0778 | -0.1907 |
| I have confidence in the robot ability to get the job done | 0.0192 | 0.2857 | -0.158 | 0.0113 | -0.0214 |
| I couldn't get anything accomplished with the robot | 0.0188 | 0.312 | 0.0258 | 0.1409 | -0.0184 |
| The vocabulary of the robot is appropriate | 0.0442 | 0.2749 | -0.1034 | -0.0446 | 0.1244 |
| The robot talks fluently | -0.1623 | 0.3104 | 0.0996 | -0.0566 | 0.2413 |
| The robot is able to manage communication failures | 0.0384 | 0.3033 | 0.2391 | -0.0612 | -0.0258 |
| How do you feel when the robot was moving his arm? quiescent-surprise | -0.0323 | 0.2619 | -0.2244 | -0.2161 | -0.0581 |
| I do not have the technical competences to make a good use of the robot | 0.0075 | 0.3296 | 0.1886 | -0.0884 | 0.0289 |
| I am willing to my living environment to be able to use the robot | 0.0622 | 0.2836 | 0.0607 | 0.023 | -0.0565 |
| The robot could be a friend of mine | 0.2227 | -0.2066 | -0.1217 | -0.1691 | 0.1275 |
| I would like to have a friendly chat with the robot | 0.2978 | 0.013 | 0.0088 | 0.0042 | -0.0651 |
| When talking with the robot I felt like I’m talking to a real person | 0.2575 | -0.0698 | 0.0322 | 0.0118 | 0.0184 |
| The robot talks fluently | 0.2123 | 0.0216 | 0.1774 | 0.0834 | -0.1637 |
| I think talk to the robot is very easy | 0.2773 | 0.0577 | -0.0182 | 0.0564 | 0.0374 |
| The robot is appealing and I really would like to use it more | 0.1906 | 0.166 | 0.0301 | 0.0376 | 0.0104 |
| I think I could have a good use of the robot | 0.2903 | 0.0077 | -0.1168 | 0.0491 | -0.014 |
| The robot is able to fulfil the goal I have setted | 0.3093 | -0.1027 | -0.015 | -0.0969 | 0.0617 |
| Robot services (reminding, obj transport, commun, ...) match the needs I have | 0.2785 | 0.0249 | 0.0077 | -0.0341 | 0.0395 |
| I have had fun using the robot | 0.1835 | 0.1054 | -0.073 | 0.0352 | 0.0198 |
